# Resting State Brain Entropy Alterations in Relapsing Remitting Multiple Sclerosis

**DOI:** 10.1371/journal.pone.0146080

**Published:** 2016-01-04

**Authors:** Fuqing Zhou, Ying Zhuang, Honghan Gong, Jie Zhan, Murray Grossman, Ze Wang

**Affiliations:** 1 Department of Radiology, the First Affiliated Hospital, Nanchang University, Nanchang, Jiangxi Province, China; 2 Department of Psychology, Hangzhou Normal University, Hangzhou, Zhejiang Province, China; 3 Jiangxi Province Medical Imaging Research Institute, Nanchang, Jiangxi Province, China; 4 Department of Oncology, the Second Hospital of Nanchang, Nanchang, Jiangxi Province, China; 5 Department of Neurology, University of Pennsylvania, Philadelphia, Pennsylvania, United States of America; 6 Zhejiang Key Laboratory for Research in Assessment of Cognitive Impairments, Hangzhou, Zhejiang Province, China; Friedrich-Alexander University Erlangen, GERMANY

## Abstract

Brain entropy (BEN) mapping provides a novel approach to characterize brain temporal dynamics, a key feature of human brain. Using resting state functional magnetic resonance imaging (rsfMRI), reliable and spatially distributed BEN patterns have been identified in normal brain, suggesting a potential use in clinical populations since temporal brain dynamics and entropy may be altered in disease conditions. The purpose of this study was to characterize BEN in multiple sclerosis (MS), a neurodegenerative disease that affects millions of people. Since currently there is no cure for MS, developing treatment or medication that can slow down its progression represents a high research priority, for which validating a brain marker sensitive to disease and the related functional impairments is essential. Because MS can start long time before any measurable symptoms and structural deficits, assessing the dynamic brain activity and correspondingly BEN may provide a critical way to study MS and its progression. Because BEN is new to MS, we aimed to assess BEN alterations in the relapsing-remitting MS (RRMS) patients using a patient versus control design, to examine the correlation of BEN to clinical measurements, and to check the correlation of BEN to structural brain measures which have been more often used in MS studies. As compared to controls, RRMS patients showed increased BEN in motor areas, executive control area, spatial coordinating area, and memory system. Increased BEN was related to greater disease severity as measured by the expanded disability status scale (EDSS) and greater tissue damage as indicated by the mean diffusivity. Patients also showed decreased BEN in other places, which was associated with less disability or fatigue, indicating a disease-related BEN re-distribution. Our results suggest BEN as a novel and useful tool for characterizing RRMS.

## Introduction

Multiple sclerosis (MS) is an inflammatory central nervous system disease that causes physical and psychiatric problems including weakness, visual loss, bowel, fatigue, mood symptoms, cognitive issues, and psychiatry problems such as depression, anxiety, etc [1,2]. MS has an early disease onset, long disease duration, and a variable disease course, making acute diagnose difficult. More importantly, the cause of MS is still not clear and there is no known cure. Current treatment research focuses on improving function after disease attack or preventing new attacks. A critical step to developing such kind of treatment or medication is to find a biomarker sensitive to disease onset and disease progression. Since the last decade, brain imaging has been increasingly used in MS to assess brain abnormality in MS. Structural MRI has been shown to be useful for detecting focal abnormalities of white matter, atrophy and monitoring disease evolution in MS [1]. But in the early course of disease, MS may not show detectable structural deficits. Alternatively, functional brain activity may carry important brain signatures sensitive to disease and its progression [1,3].

Functional brain alterations have been studied in MS using the task-evoked activity. But MS is a slowly progressive neurodegenerative process with minimal apparent clinical symptom in the initial phase [1,2,4]. Such “silent” degenerations may not become overt dysfunctions detectable by functional MRI (fMRI) through the task-invoked brain activation. Meanwhile, task performance only causes less than increase 5% energy consumption. By contrast, the major component of brain activity, spontaneous brain activity (SBA), accounts for most of brain energy consumption [5], and assessment of this may provide a more sensitive way to detect MS-related functional alterations. SBA is widely assessed using resting-state fMRI (rs-fMRI). Several SBA features, such as functional connectivity (FC), the amplitude of low-frequency fluctuations (ALFF) [6], have been examined in MS [7,8]. But the findings were somewhat inconsistent to each other. While these discrepancies may reflect an inhomogeneity of disease stages or population variability, they may also reflect a technical need for a more comprehensive SBA measure rather than those empirical ones used in those studies.

Entropy is a statistical and physical index that measures the irregularity, and thereafter the health of a dynamic system [9]. Higher entropy indicates increased randomness of a system, meaning the dynamic system activity is less predictable and less organized. Using electrophysiological data, entropy has long been used for assessing different brain states [10–14]. With fMRI, we have recently demonstrated reliable and spatially distributed brain entropy (BEN) patterns in normal brain [15,16]. The normal BEN distributions, however, may be altered in disease conditions. Indeed, BEN alterations have been demonstrated in aging [17], schizophrenia [18] and attention deficit hyperactivity disorder (ADHD) [19]. In this study, we aimed to explore BEN alterations in relapsing-remitting MS (RRMS) patients compared to age-matched healthy controls. Since regional BEN reflects the dynamic state of the brain, it cannot be fully characterized using any other methods. To evaluate BEN for MS, we collected rsfMRI data, structural MRIs, and clinical measures from RRMS, which is in the early stage of MS. As an initial study, we expected to see MS-related BEN alterations and their correlations with micro-structural brain deficits, and disease-related clinical measures.

## Materials and Methods

All study procedures adhered to the Declaration of Helsinki and were approved by the Institutional Review Board of the First Affiliated Hospital, Nanchang University, China. All of the subjects provided written informed consent before participating in any study procedure.

### Subjects

Thirty-four right-handed RRMS patients and 34 right-handed sex-, age-, and education level-matched healthy subjects were recruited from the First Affiliated Hospital and local community, respectively. All patients were diagnosed to be definite MS based on the McDonald’s criteria [20], with clinical evidence of relapsing remitting course [20]. Patients were currently treated with immunomodulatory medication (29 with β-interferon, 5 with Glatiramer acetate). None of them had any relapses or cortico-steroid treatment during the month preceding the MR acquisition. The clinical and demographic data are listed in Table 1. Disability was characterized with the expanded disability status scale (EDSS). Functional impairment faced by the patients were evaluated in terms of processing speed and fatigue using the paced auditory serial addition test (PASAT) and the modified fatigue impact scale 5 (MFIS-5), respectively.

**Table 1:** Demographic and clinical information of the study population.

	RRMS patients (remitting phase)	Healthy controls	P values
Gender (M/F)	13/21	13/21	> 0.99
Mean age (range) (years)	42.1 (20–58)	41.8 (21–58)	0.96
Mean disease duration (range) (months)	27.10 (1.5–150)	n/a	n/a
Median EDSS (range)	2.06(0–4)	0	n/a
Mean PASAT (range)	84.13 (61–103)	97.82(82–118)	0.001
Mean MFIS-5 (range)	11.29 (6–17)	0.29 (0–1)	0.000
Mean TWMLL (range) (ml) (normalized)	18.33(0.43–79.41)	n/a	n/a
Mean BPF (range)	0.829 (0.78–0.87)	0.86(0.82–0.88)	0.000

Note: BPF = brain parenchymal fraction, EDSS = expanded disability status scale; F = female; M = male; MFIS = modified fatigue impact scale; n/a = not applicable; PASAT = paced auditory serial addition test; RRMS = relapsing-remitting multiple sclerosis, TWMLL = total white matter lesion load.

### Data acquisition

The following MRI scans were performed on a Trio 3.0 Tesla MRI scanner (Siemens, Erlangen, Germany): 1) rs-fMRI with repetition time (TR)/echo time (TE) = 2000/30 ms, field of view (FOV) = 200 × 200 mm^2^, matrix = 64 × 64, 30 interleaved axial slices with thickness = 4 mm and inter-slice gap = 1.2 mm. 240 time points were acquired; 2) High resolution anatomical scan using a three dimensional magnetization prepared rapid gradient echo (MPRAGE) sequence with the following parameters: TR/TE = 1900/2.26 ms; matrix = 240 × 256 × 176; FOV = 215 × 230 × 176 mm^3^; 3) Diffusion tensor imaging (DTI) with TR/TE = 7200/104 ms; matrix = 128 × 128; FOV = 230 × 230 mm^2^; 49 axial slices (thickness = 2.5 mm); 64 nonlinear gradient directions (b = 0, 1000 s/mm^2^); 4) Two dimensional structural MRI with a T2-weighted sequence with TR/TE = 5100/117 ms; matrix = 416×416; FOV = 240×240 mm^2^; number of excitations = 3; 22 axial slice with a thickness of 6.5 mm.

### Image pre-processing and BEN mapping

rsfMRI images were preprocessed using Data Processing Assistant for Resting-State fMRI Advanced Edition (DPARSFA) V2.3 (http://rfmri.org/DPARSF_V2_3), which is based on SPM8 (http://www.fil.ion.ucl.ac.uk/spm/software/spm8/) and Matlab 7.14.0 (Mathworks, Natick, MA, USA). The same processing steps as in our previous study [21] were included: 1) discarding the first 10 images to allow signal to reach stead state; 2) slice-timing correction and motion correction; 3) registering rsfMRI to the Montreal Neurological Institute (MNI) standard brain space via each subject’s T1 weighted anatomical image and resampling them to 3-mm cube voxels. The high resolution structural MRI-based spatial registration was performed using the diffeomorphic anatomical registration through exponentiated lie algebra (DARTEL) algorithm provided in SPM8 [22]; 4) spatial smoothing with an isotropic Gaussian kernel (full-width-at-half-maximum (FWHM) = 6-mm^3^); 5) linearly detrending and temporal band-pass filtering (0.01–0.08 Hz) to eliminate high-frequency noise and low-frequency drift; 6) temporal nuisance correction using simple regression with the residual head motions, white matter signal, and cerebrospinal fluid (CSF) signal as the co-variants [23]. Subjects were excluded from further analyses if their mean head motions were greater than 2 mm in translations along any the 3 orthogonal axes and less than 2.0° in the 3 directional angular rotations.

After pre-processing, BEN mapping was performed using the Brain Entropy mapping toolbox [15] (https://cfn.upenn.edu/~zewang/BENtbx.php). BEN was calculated at each voxel Sample Entropy (SampEn) [24]. SampEn is an approximate entropy measure, which measures the temporal coherence of a time series through calculating the “logarithmic likelihood” that a small section (within a window of a length ‘m’) of the data “matches” with other sections will still “match” the others if the section window length increases by 1. “match” is defined by a threshold < ‘*r*’ times standard deviation of the entire time series. Based on our previous work, the window length was set to 3 and the cut off threshold was set to 0.6 [15]. Denoting the rsfMRI data of one voxel by *x* = [*x*_1_,*x*_2_,…*x*_*N*_], where *N* is the number of acquisitions, SampEn calculation starts with forming a series of vector sequences, each with *m* consecutive points extracted from *x*: *u*_*i*_ = [*x*_*i*_, *x*_*i*_ + 1,…*x*_*i*_ + *m* − 1], where *i* = 1 to *N* − *m* + 1, and m is the pre-defined window size (3, as stated above). Using a pre-specified distance threshold *r* (= 0.6), the number of embedded vectors *u*_*j*_(*j* = 1, to *N* − *m*, and *j* ≠ *i*) whose distance from *u*_*j*_ are less than *r* is recorded by $B_{i}^{m}\left(r\right)$. The same procedure is repeated for the dimension of *m* + 1 to get $B_{i}^{m+1}\left(r\right)$. By averaging across all possible vectors, we can get $B^{m}\left(r\right)=\frac{1}{\left(N-m\right)\left(N-m+1\right)}\sum_{i=1}^{N-m}B_{i}^{m}\left(r\right)$, $A^{m}\left(r\right)=\frac{1}{\left(N-m\right)\left(N-m-1\right)}\sum_{i=1}^{N-m}B_{i}^{m+1}\left(r\right)$, and SampEn is calculated as: $SampEn\left(m,r,N,x\right)=-\ln\left[\frac{A^{m}\left(r\right)}{B^{m}\left(r\right)}\right]$.

The collection of all voxels’ entropy values formed the BEN map, which was smoothed with an isotropic Gaussian kernel (FWHM = 6mm^3^). A Fisher’s z-transform was applied to the BEN maps before performing group level analysis.

### Diffusivity measure calculations

DTI data were processed using Functional MRI of the Brain (FMRIB's) diffusion toolbox (fdt-v2.0, http://fsl.fmrib.ox.ac.uk/fsl/FDT). Processing steps included image distortion correction, motion correction, and spatial smoothing. Both fractional anisotropy (FA) and mean diffusivity (MD) were calculated. The consequent FA and MD maps were registered with T1 image using the b0 map as the registration reference, and were spatially normalized into MNI space using the Non-linear Image Registration Tool (FNIRT) provided in FMRIB Software Library (FSL).

### Lesion load and brain atrophy evaluations

Lesion load was assessed using the total white matter lesion load (TWMLL) [25,26]. The procedures for calculating TWMLL were the same as described in [25]. All visible lesions were identified from the T2 weighted image and a binary lesion mask was manually drawn with MRIcron (http://www.nitrc.org/projects/mricron) by a radiologist (F.Z). The T2 weighted image was coregistered with the T1 weighted structural image. The aforementioned T1 image-based individual brain to MNI standard brain transform was used to warp the lesion mask into the MNI space. The lesion load calculated from the spatially normalized lesion mask reflects the TWMLL in relative to the standard brain volume rather than the individual brain volume so that the effects of different brain volume were controlled. Lesions were re-measured on two separate occasions (at least three months apart) in the patients, and the inter-rater reliability was 92.6%.

T1-weighted structural image was segmented into gray matter (GM), white matter (WM), CSF using the new segmentation algorithm provided in SPM8. The GM and WM probability images were then registered and warped into MNI space using the aforementioned DARTEL process. The brain parenchymal fraction (BPF) was then calculated as the ratio of brain parenchymal (GM and WM) volume to the intracranial volume.

### Statistical analysis

Cross-sectional BEN difference (RRMS vs controls) was assessed using a two-sample t-test implemented in SPM8. Age, gender, and education level were included as nuisances. Statistical inference was made with a threshold of p < 0.01 at the voxel-level and a cluster size > 50 at the cluster level. The cluster level p-value was determined using the Monte Carlo simulation-based multiple comparison correction implemented in the AlphaSim program in Analysis of Functional NeuroImages (AFNI). 1000 permutations were performed.

Correlations between regional BEN and clinical symptoms (measured by EDSS and MFIS-5) or DTI measures were examined using simple regressions in Statistical Product and Service Solutions (SPSS version 13, Chicago, IL, USA). Mean BEN values were extracted from the suprathreshold clusters identified in the above cross-sectional analysis. Significance level was set be p < 0.05 with Bonferroni correction.

To validate the clinical implications of BEN vs clinical measure correlations, a leave-one-out cross-validation was performed. At each validation, a prediction model was built using simple regression based on the clinical measures and BEN values from n-1 (n = 34) patients. The clinical symptom of the remained one subject was then predicted by feeding his/her BEN value into the prediction model. The same process was then repeated by picking up a new subject as the testing subject and the remained n-1 as the training samples until all subjects were tested. The predicted values for all subjects were then correlated with the true values, and the correlation coefficient was used as a prediction accuracy index of BEN for clinical measures.

### ALFF analysis

Since BEN is still new in the literature, we calculated ALFF and repeated the above mentioned group level analysis as a comparison. ALFF [6,27] was chosen because it is a regional SBA measure and it has been widely used in resting state studies [27]. Each voxel’s time series was transformed into the frequency domain using Fourier transform, and ALFF was calculated as the root of the sum of square of the magnitude of the frequency spectrum within the range of 0.01–0.08 Hz. The ALFF map was subsequently transformed into z-scores using the Fisher's r-to-z transformation.

## Results

TWMLL and BPF calculation results were listed in Table 1. BPF was significantly lower in RRMS than in controls.

### Mean BEN maps

S1 Fig shows the mean BEN maps of the RRMS patients and healthy controls. Both maps showed a clear entropy contrast between GM and WM with lower entropy in GM.

### Altered BEN in RRMS

**Fig 1:**
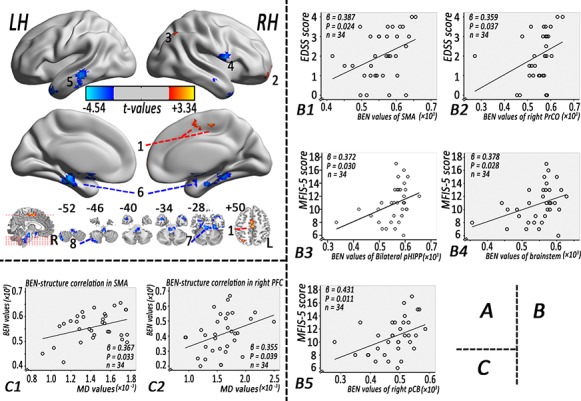
Altered BEN in the RRMS patients and its correlations to disability and structural measures. (A) BEN difference between the RRMS patients and healthy control. Red and blue colors indicate increased and decreased BEN, respectively. The color bars indicate the display window for t-values. (B) Correlations of BEN in the RRMS patients to clinical measures. (C) BEN correlations with MD in the RRMS patients. Note: BEN: brain entropy, EDSS: expanded disability status scale, LH: left hemisphere, MD: mean diffusivity, RH: right hemisphere, pCB: posterior cerebellum, pHIPP: parahippocampal gyrus, PrCO: precentral operculum, PFC: prefrontal cortex, RRMS = relapsing-remitting multiple sclerosis, SMA: supplementary motor area.

BEN comparison results were shown in Fig 1A and Table 2. As compared to controls, RRMS patients showed significantly increased BEN in bilateral supplementary motor area (SMA), right prefrontal cortex (PFC), and right angular gyrus, but decreased BEN in right precentral operculum (PrCO), left middle temporal gyrus (MTG), bilateral parahippocampus (pHIPP), brainstem, and right posterior cerebellum (pCB).

**Table 2:** Brain areas of altered BEN in the RRMS patients compared with the healthy controls, p < 0.05, corrected for AlphaSim, cluster size ≥ 50 voxels.

Cluster site		Peak MNI coordination (x,y,z)	Peak intensity (t values)	Cluster size (voxel)
RRMS > Healthy control				
1	Bilateral supplementary motor area (SMA)	0, -21, 51	2.96	79
2	Right prefrontal cortex (PFC)	15, 69, -3	3.43	53
3	Right angular gyrus	30, -69, 51	2.63	55
RRMS < Healthy control				
4	Right precentral operculum (PrCO)	57, 3, 12	-3.35	86
5	Left middle temporal gyrus (MTG)	-60, -27, -12	-3.19	127
6	Bilateral parahippocampal gyrus (pHIPP)	-30, -27, -18	-3.93	627
7	Brainstem	-6, 0, -18	-4.54	566
8	Right posterior cerebellum (pCB)	42, -69, -51	-3.44	118

Note: MNI = Montreal Neurological Institute; RRMS = relapsing-remitting multiple sclerosis.

### Clinical associations of BEN in RRMS

Fig 1B and S1 Table show the BEN vs clinical measure correlations. BEN in RRMS patients was correlated with EDSS in bilateral SMA (R^2^ = 0.150, β = 0.387, p = 0.024) and PrCO (R^2^ = 0.129, β = 0.359, p = 0.037), and correlated with the MFIS-5 scores in bilateral pHIPP (R^2^ = 0.138, β = 0.372, p = 0.030), brainstem (R^2^ = 0.143, β = 0.378, p = 0.028) and right pCB (R^2^ = 0.186, β = 0.431, p = 0.011). The accuracy of using bilateral SMA BEN to predict EDSS, using PrCO BEN to predict EDSS, using bilateral pHIPP BEN for predicting MFIS-5, brainstem BEN for MFIS-5, and right pCB BEN for MFIS-5 were R^2^ = 0.018 with p = 0.447, R^2^ = 0.116 with p = 0.048, R^2^ = 0.127 with p = 0.039, R^2^ = 0.134 with p = 0.034, and R^2^ = 0.141 with p = 0.029, respectively.

### BEN-structure correlations in RRMS

Fig 1C and S2 Table show the correlations between BEN and DTI measures in RRMS. BEN was significantly correlated with local MD values in bilateral SMA (R^2^ = 0.135, p = 0.033, β = 0.367) and right PFC (R^2^ = 0.126, p = 0.039, β = 0.355); BEN in bilateral SMA (R^2^ = 0.124, p = 0.041) and right PFC (R^2^ = 0.126, p = 0.039) predicted local microstructure tissue damage as measured by MD. No significant relations were found between BEN and local FA values (p: 0.152 to 0.973), brain parenchymal fraction (p: 0.149 to 0.870), or T2 lesion volume (p: 0.057 to 0.996).

### ALFF analysis results

**Fig 2:**
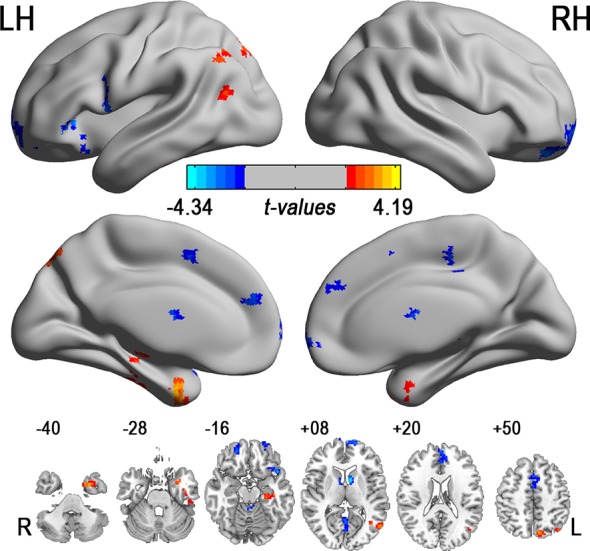
Altered ALFF in the RRMS patients. Note: ALFF: amplitude of low frequency fluctuation, L: left, LH: left hemisphere, R: right, RH: right hemisphere; RRMS = relapsing-remitting multiple sclerosis. The color bars indicate the display window for t-values.

As a comparison, Fig 2 and S3 Table show the results of ALFF-based analyses. RRMS patients showed lower ALFF in frontal cortex but higher ALFF in temporal, parietal and visual cortex. ALFF in RRMS patients was negatively correlated with EDSS in bilateral pHIPP (R^2^ = 0.118, β = -0.344, p = 0.046), left pHIPP (R^2^ = 0.137, β = -0.370, p = 0.031), bilateral superior frontal gyrus (R^2^ = 0.136, β = -0.403, p = 0.018), and bilateral PFC (R^2^ = 0.139, β = -0.372, p = 0.030). However, there were no significant relations between ALFF and other clinical measures (p: 0.206 to 0.943).

## Discussion

Resting BEN was assessed in RRMS using brain entropy mapping. BEN is a new metric recently proposed for characterizing resting state brain activity [15,16, 28–30]. Different from other resting state metrics such as FC, resting state networks, regional homogeneity, ALFF, etc, BEN indicates the state of the temporal brain dynamics, measuring the irregularity of brain activity. Higher irregularity means higher unpredictability, which may correspond to higher vulnerability to internal or external disruptions. In this study, we found both increased and decreased resting BEN in RRMS in areas involved in motor control, integration and execution (SMA, PrCO, right pCB, and brainstem), cognitive control (PFC), spatial coordination (angular gyrus), and memory (MTG and pHIPP) function. While we don’t have data showing functional alterations in the BEN-affected brain regions when patients are explicitly challenged with a functional test, these regions have been identified in previously published functional or structural brain studies in MS [31,32]. BEN alterations in those areas suggest an altered functional temporal dynamics of the resting brain in RRMS, providing new and complimentary information to functional brain differences in RRMS reported by the literature.

Increased BEN in SMA, angular gyrus, and PFC indicates more irregular (disordered) brain activity therein than other locations. Since SMA, angular gyrus, and PFC are associated with motor planning, spatial coordinating, and cognitive control, increased BEN in those regions is consistent with the major symptoms of having difficulty of motion and spatial coordination, and cognitive dysfunction in MS patients. In SMA, we further found a positive correlation between BEN and EDSS, suggesting that BEN alteration in SMA is directly related with RRMS with higher BEN corresponding to more severe disability. Using cross-validations, we further showed that BEN is linearly predictive of functional impairments of RRMS patients, though the BEN vs EDSS linear association didn’t survive cross-validations.

Reduced BEN was also observed in several brain regions, including PrCO, MTG, pHIPP, brainstem, and pCB, indicating more regular resting state activity in RRMS in those regions. Increased regularity in RRMS might represent a compensation mechanism [33] for the BEN increase in the aforementioned areas when MS is still in the early stage (RRMS), which was further supported by our clinical implication analysis results. By relating BEN to clinical measures, we found that lower BEN in PrCO was related with less severe disease (EDSS); lower BEN in pHIPP, rpCB, and brainstem was associated with lower fatigue level, a typical index for the functional impairment suffered by MS patients. These data suggest that the compensation might be manifested as a redistribution of BEN, meaning that some brain areas become more regulated (or equivalently more temporally coherent) in order to compensate the reduced regularity in the disease target area such as SMA.

To examine BEN’s connections with structural brain measures, we calculated its correlations with several structural measures. Higher BEN was found to be associated with higher level of local tissue damage (higher MD, S4 Table) in SMA and right PFC, suggesting that tissue damage contributes to increase BEN in RRMS. Increased MD is typical in MS though might be benign in RRMS [34]. Since MD is mainly dependent on the free space water, increased MD may indicate axonal or myelin lesion or edema, which can all cause alterations to the regularity of brain activity because those changes damage the physical inter-neuronal connections. Nevertheless, the BEN-MD correlations proved that BEN is a meaningful brain index that coupled with a structural brain measure.

We also compared BEN with a frequency analysis-based method, ALFF and found that ALFF alteration patterns in RRMS only showed minor overlap with BEN alteration patterns in temporal cortex. This difference clearly indicates that BEN provides a different view of brain status that cannot be fully characterized by only examining a certain frequency band of the entire signal (note that ALFF focuses on the low frequency band only). In the clinical measure correlation analyses, ALFF only showed correlations to EDSS not to MFIS-5. These differences suggest that BEN provides more comprehensive information about RRMS that cannot be characterized by ALFF.

Several limitations were in the present study. First, we only studied the brain. Spinal cord lesion is one key factor of MS that has been shown to be highly predictive of disability in MS [35]. Future study may need to include that. Second, BEN calculation was based on SampEn in this study, which has two parameters that may yield affect the estimated entropy but should not affect the group-level entropy comparison results, as supported by additional analyses with various different SampEn parameters (see S2 Fig).

## Conclusion

As an initial BEN mapping study in RRMS, our data suggest that MS disease-related functional brain changes can be detected with BEN; BEN alterations reflect disease severity, functional impairment, as well as regional micro-structural damages. Our findings suggest BEN as a potential marker for associating MS-related disability, fatigue, or tissue damage, though these findings should be re-assessed using independent cohorts or test-retest data.

## Supporting Information

S1 Dataset Brain entropy analysis of RRMS patients and HC groups. (ZIP)

S2 Dataset ALFF analysis of RRMS patients and HC groups. (ZIP)

S1 Fig Whole brain entropy maps of healthy control (A) and the RRMS (B) group (one sample t-test) in different pre-specified distance threshold (r values). Warm and cold colors denote higher and lower than the mean of whole brain, respectively. Images are displayed in radiological convention.
(TIF)

S2 Fig Map wise brain entropy (BEN) alterations in the RRMS patients (vs. healthy control) in different pre-specified distance threshold (r values), p < 0.05, cluster size *≥* 50 voxels (1350 mm^3^), with corrected. Red and blue colors denote increased and decreased BEN, respectively. The color bars indicate the t-values. Images are displayed in radiological convention. Exemplary the BEN values altered pattern of the left pHIPP (F) and the bilateral SMA (G) in different pre-specified distance threshold (r values). The broken line means the compared t values of two groups in different pre-specified distance threshold (r values). For examples, the masks of the left pHIPP and the bilateral SMA were extracted based on the union of the altered BEN maps from the different pre-specified distance threshold conditions.
(TIF)

S1 Table Correlation between BEN and the RRMS clinical measures. (DOC)

S2 Table The relationship of the MS-related BEN-structural coupling measures. (DOC)

S3 Table Brain areas of altered ALFF in the RRMS patients compared with the healthy controls, p < 0.05, corrected for AlphaSim, cluster size ≥ 50 voxels. (DOC)

S4 Table Measure analyses of local DTI variance corresponding regions with abnormal BEN values. (DOC)
