# Supplementary figures and images for: Resting State Brain Entropy Alterations in Relapsing Remitting Multiple Sclerosis
Source: PLoS One. 2016 Jan 4;11(1):e0146080. doi: 10.1371/journal.pone.0146080 (PMC4699711; doi:10.1371/journal.pone.0146080)

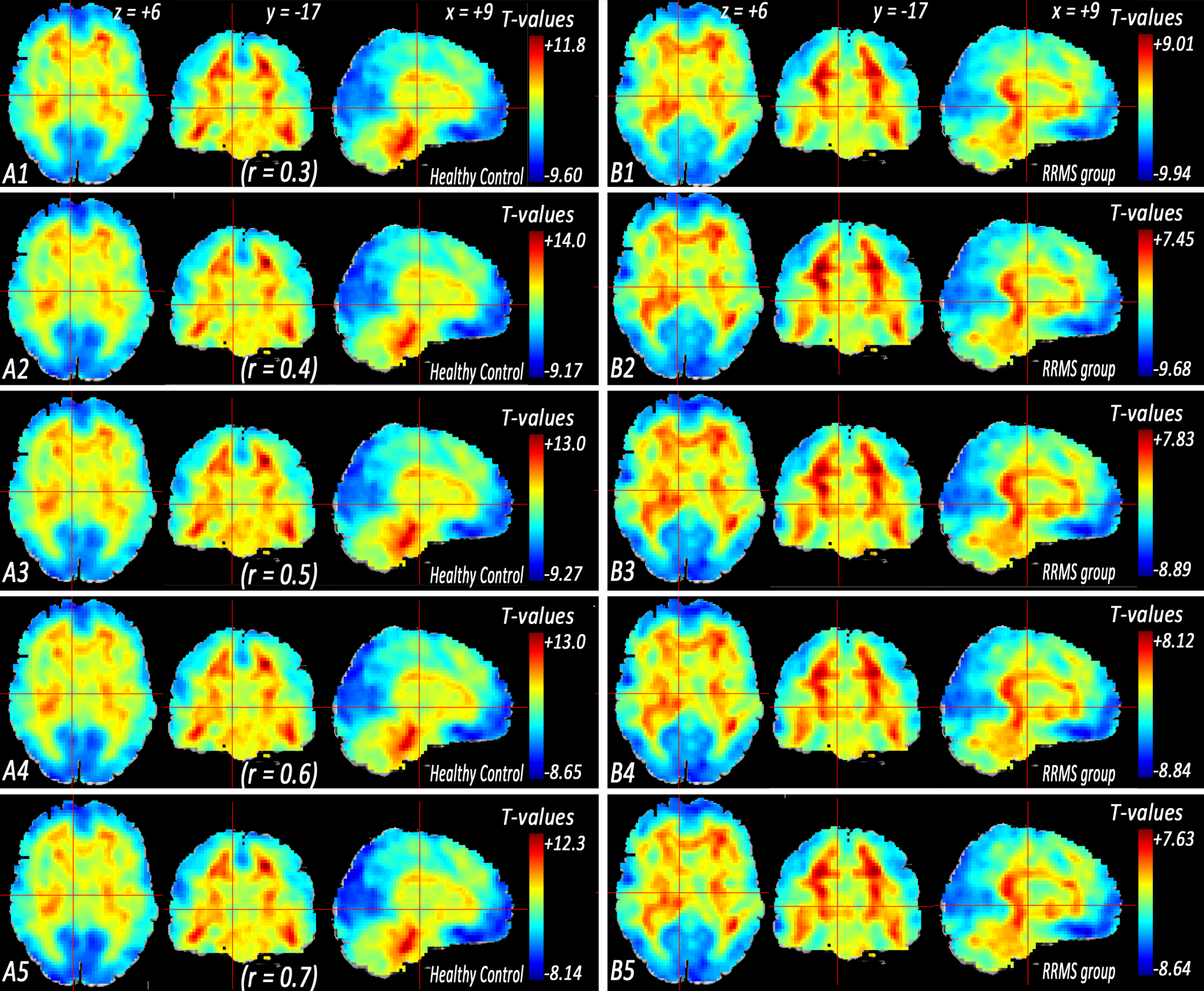

Supplement: S1 Fig — Warm and cold colors denote higher and lower than the mean of whole brain, respectively. Images are displayed in radiological convention. (TIF) [file pone.0146080.s003.tif]

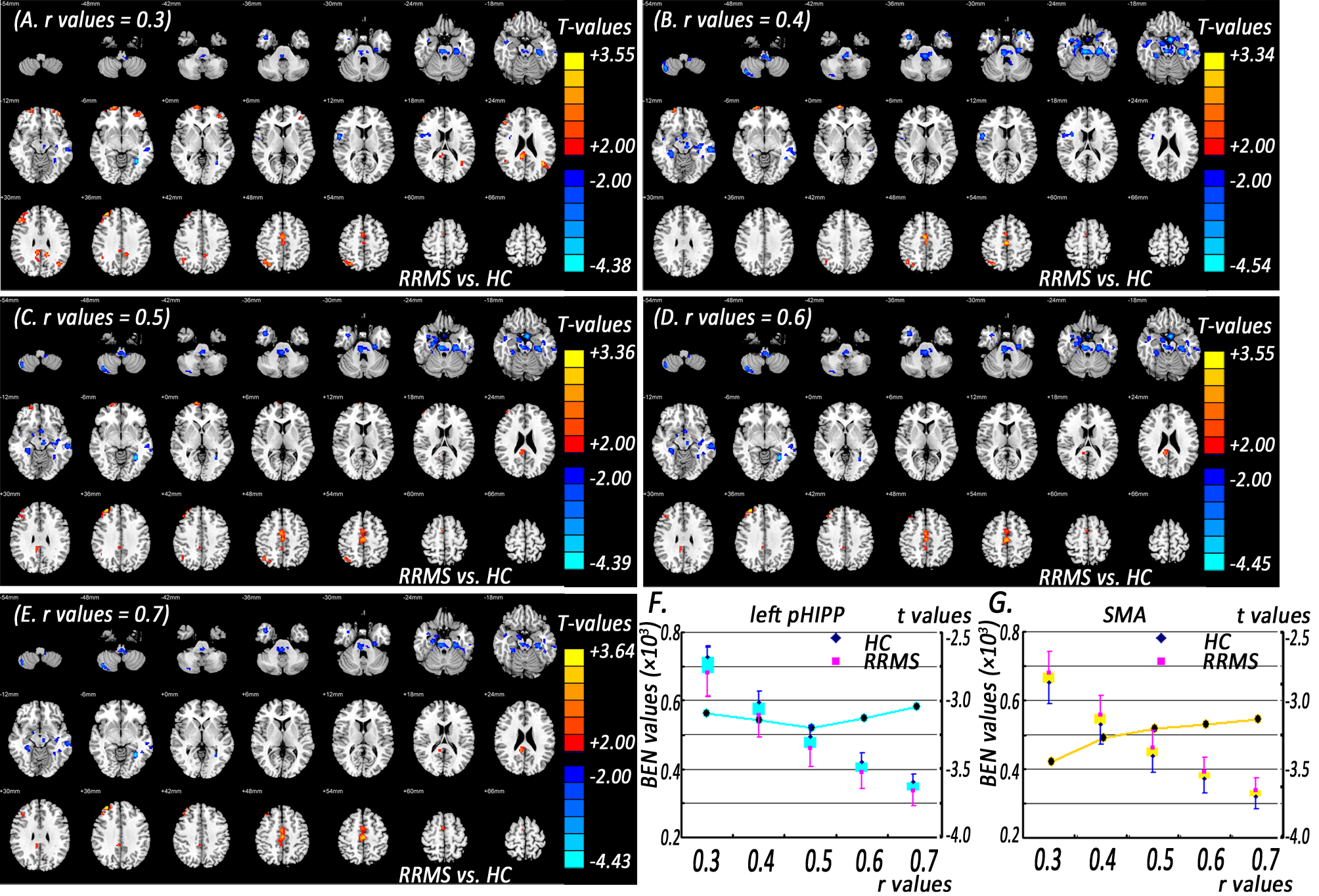

Supplement: S2 Fig — Red and blue colors denote increased and decreased BEN, respectively. The color bars indicate the t-values. Images are displayed in radiological convention. Exemplary the BEN values altered pattern of the left pHIPP (F) and the bilateral SMA (G) in different pre-specified distance threshold (r values). The broken line means the compared t values of two groups in different pre-specified distance threshold (r values). For examples, the masks of the left pHIPP and the bilateral SMA were extracted based on the union of the altered BEN maps from the different pre-specified distance threshold conditions. (TIF) [file pone.0146080.s004.tif]
