# Supplementary material for: Resting State Brain Entropy Alterations in Relapsing Remitting Multiple Sclerosis
Source: PLoS One. 2016 Jan 4;11(1):e0146080. doi: 10.1371/journal.pone.0146080 (PMC4699711; doi:10.1371/journal.pone.0146080)
Supplement: S1 Table — (DOC) [file pone.0146080.s005.doc]

S1 Table Correlation between BEN and the RRMS clinical measures.

|  | disease duration | | EDSS | | PASAT | | MFIS-5 | |
| --- | --- | --- | --- | --- | --- | --- | --- | --- |
|  | *β* | *P* | *β* | *P* | *β* | *P* | *β* | *P* |
| Bilateral SMA | 0.079 | 0.685 | **0.387** | **0.024** | 0.264 | 0.166 | 0.120 | 0.500 |
| Right PFC | 0.224 | 0.243 | 0.114 | 0.526 | 0.102 | 0.598 | 0.127 | 0.474 |
| Right angular gyrus | -0.063 | 0.744 | 0.196 | 0.266 | 0.017 | 0.929 | 0.165 | 0.350 |
| Right PrCO | 0.088 | 0.650 | **0.359** | **0.037** | 0.084 | 0.666 | 0.337 | 0.051 |
| Left MTG | 0.038 | 0.847 | 0.241 | 0.171 | 0.003 | 0.988 | 0.237 | 0.176 |
| Bilateral pHIPP | 0.114 | 0.556 | 0.257 | 0.143 | 0.013 | 0.948 | **0.372** | **0.030** |
| brainstem | 0.260 | 0.173 | 0.202 | 0.251 | 0.005 | 0.980 | **0.378** | **0.028** |
| rpCB | 0.254 | 0.183 | 0.260 | 0.173 | 0.169 | 0.381 | **0.431** | **0.011** |

Note: EDSS = expanded disability status scale; MFIS = modified fatigue impact scale; PASAT = paced auditory serial addition test; SMA = supplementary motor area; PFC = prefrontal cortex; PrCO = precentral operculum; MTG = middle temporal gyrus; pHIPP = parahippocampus gyrus; rpCB = right posterior cerebellum; RRMS = relapsing-remitting multiple sclerosis.
