# Supplementary material for: Resting State Brain Entropy Alterations in Relapsing Remitting Multiple Sclerosis
Source: PLoS One. 2016 Jan 4;11(1):e0146080. doi: 10.1371/journal.pone.0146080 (PMC4699711; doi:10.1371/journal.pone.0146080)
Supplement: S2 Table — (DOC) [file pone.0146080.s006.doc]

S2 Table The relationship of the MS-related BEN-structural coupling measures.

|  | *Local FA | | Local MD | | TWMLL | | BPF | |
| --- | --- | --- | --- | --- | --- | --- | --- | --- |
|  | β | P | β | P | β | P | β | P |
| Bilateral SMA | 0.006 | 0.938 | 0.367 | 0.033 | 2.182 | 0.149 | 0.898 | 0.352 |
| Right PFC | 1.071 | 0.309 | 0.355 | 0.039 | 0.085 | 0.773 | 0.002 | 0.962 |
| Right angular gyrus | 0.140 | 0.711 | 0.083 | 0.775 | 0.813 | 0.374 | 3.965 | 0.057 |
| Right PreG | 0.001 | 0.973 | 1.124 | 0.297 | 1.779 | 0.192 | 0.000 | 0.996 |
| Left MTG | 0.000 | 0.988 | 0.566 | 0.458 | 0.638 | 0.430 | 0.385 | 0.540 |
| Bilateral pHIPP | 0.165 | 0.688 | 0.462 | 0.502 | 1.246 | 0.273 | 0.354 | 0.557 |
| Brainstem | 0.137 | 0.714 | 0.447 | 0.509 | 1.015 | 0.321 | 3.604 | 0.069 |
| Right CPL | 2.152 | 0.152 | 0.026 | 0.874 | 0.027 | 0.870 | 0.870 | 0.360 |

Note: *Local DTI values are the corresponding regions with abnormal BEN values. FA = fractional anisotropy; MD = mean diffusivity; AD = axial diffusivity; RD = radial diffusivity; SMA = supplementary motor area; SFG = superior frontal gyrus; PreG = precentral gyrus; MTG = middle temporal gyrus; pHIPP = parahippocampal gyrus; CPL = cerebellum posterior lobe.
