# Supplementary material for: Resting State Brain Entropy Alterations in Relapsing Remitting Multiple Sclerosis
Source: PLoS One. 2016 Jan 4;11(1):e0146080. doi: 10.1371/journal.pone.0146080 (PMC4699711; doi:10.1371/journal.pone.0146080)
Supplement: S3 Table — (DOC) [file pone.0146080.s007.doc]

S3 Table Brain areas of altered ALFF in the RRMS patients compared with the healthy controls, *P* < 0.05, corrected for AlphaSim, cluster size ≥ 50 voxels.

| Cluster site | | Peak MNI  coordination (x,y,z) | Peak intensity  (t values) | Cluster size (voxel) |
| --- | --- | --- | --- | --- |
| RRMS > Healthy control | | | | |
| 1 | Bilateral parahippocampal gyrus (pHIPP) | -33, 3, -30 | 3.83 | 82 |
| 2 | Left pHIPP | -39. -21, -15 | 3.10 | 74 |
| 3 | Left middle temporal gyrus | -57, -66, 12 | 3.78 | 83 |
| 4 | Left precuneus/ inferior parietal lobule/ angular gyrus | -39, -69, 48 | 3.91 | 211 |
| RRMS < Healthy control | | | | |
| 5 | Left inferior frontal gyrus | -45, 18, -9 | -4.34 | 127 |
| 6 | Bilateral superior frontal gyrus | 12, 63, -6 | -3.29 | 284 |
| 7 | Bilateral caudate | -9, 6, 9 | -4.17 | 80 |
| 8 | Bilateral lingual/ posterior cingulate cortex | 3, -39, -9 | -4.02 | 200 |
| 9 | Bilateral prefrontal cortex | -3, 57, 24 | -3.49 | 73 |
| 10 | Bilateral supplementary motor area | 0, 15, 51 | -2.82 | 76 |

Note: ALFF = amplitude of low-frequency fluctuations; MNI = Montreal Neurological Institute; RRMS = relapsing-remitting multiple sclerosis.
