# Supplementary material for: Resting State Brain Entropy Alterations in Relapsing Remitting Multiple Sclerosis
Source: PLoS One. 2016 Jan 4;11(1):e0146080. doi: 10.1371/journal.pone.0146080 (PMC4699711; doi:10.1371/journal.pone.0146080)
Supplement: S4 Table — (DOC) [file pone.0146080.s008.doc]

S4 Table. Measure analyses of local DTI variance corresponding regions with abnormal BEN values.

|  | *Local FA values | | | Local MD values (×10^-3^mm^2^/s) | | |
| --- | --- | --- | --- | --- | --- | --- |
|  | HC (mean±SE) | RRMS(mean±SE) | t (P) | HC(mean±SE) | RRMS(mean±SE) | t (P) |
| Bilateral SMA | 0.190±0.0041 | 0.182±0.0039 | 1.551 (0.126) | 1.314±0.0321 | 1.419±0.0362 | -2.170 (0.034) |
| Right PFC | 0.161±0.0045 | 0.139±0.0042 | 3.613 (0.001) | 1.496±0.0596 | 1.683±0.0635 | -2.144 (0.036) |
| Right angular gyrus | 0.280±0.0091 | 0.282±0.0105 | -0.144 (0.886) | 0.833±0.0114 | 0.904±0.0272 | -2.409 (0.019) |
| Right PreG | 0.351±0.0056 | 0.316±0.0092 | 3.238 (0.002) | 0.841±0.0116 | 0.920±0.0299 | -2.465 (0.016) |
| Left MTG | 0.176±0.0179 | 0.165±0.0054 | 0.601 (0.552) | 0.798±0.0213 | 0.933±0.0457 | -2.657 (0.011) |
| Bilateral pHIPP | 0.272±0.0081 | 0.250±0.0044 | 2.365 (0.022) | 1.062±0.0196 | 1.106±0.0234 | -1.430 (0.158) |
| Brainstem | 0.480±0.0144 | 0.466±0.0057 | 0.939 (0.353) | 0.895±0.0188 | 0.951±0.0223 | -1.913 (0.060) |
| Right CPL | 0.183±0.0057 | 0.179±0.0095 | 0.314 (0.755) | 0.824±0.0183 | 0.903±0.0442 | -1.648 (0.104) |

Note: *Local DTI values are the corresponding regions with abnormal BEN values. FA = fractional anisotropy; MD = mean diffusivity; AD = axial diffusivity; RD = radial diffusivity; SE = standard error; SMA = supplementary motor area; SFG = superior frontal gyrus; PreG = precentral gyrus; MTG = middle temporal gyrus; pHIPP = parahippocampal gyrus; CPL = cerebellum posterior lobe.
